# Editorial: Therapeutic mechanism of osteosarcoma

**DOI:** 10.3389/fmolb.2025.1597144

**Published:** 2025-04-08

**Authors:** Siyi Chen, Yang Wang

**Affiliations:** Department of Emergency Medicine Center, Sichuan Provincial People’s Hospital, University of Electronic Science and Technology of China, Chengdu, China

**Keywords:** osteosarcoma, immunotherapy, Wnt/β-catenin signaling, tumor microenvironment, CAR-T cell therapy, metabolic reprogramming

Osteosarcoma (OS) is a highly aggressive primary bone cancer with a particularly poor prognosis in metastatic cases, and treatment continues to face significant challenges due to tumor heterogeneity, immune resistance, and an unfavorable tumor microenvironment (TME) (Sabit et al., 2025). Despite important advancements in molecular biology, immunotherapy, and metabolic interventions, the treatment of OS remains difficult, requiring further research to optimize targeted therapies and improve immunotherapy and metabolic strategies. This review aims to explore the latest advancements in OS research, focusing on molecular targets, immunotherapy, metabolic reprogramming, and clinical trial strategies. Studies have shown that key signaling pathways, such as Wnt/β-catenin and mesenchymal-epithelial transition (MET), play crucial roles in OS progression, particularly in the activation at the tumor-stroma interface and the overexpression of the MET receptor. Emerging biomarkers like apo-transcobalamin-II (APO-TCN2) and death receptor 5 (DR5) have been identified, offering potential prognostic value. Additionally, chimeric antigen receptor T-cell (CAR-T) therapy and combination immunotherapies show promise in overcoming immune evasion mechanisms. Metabolic reprogramming, particularly through long non-coding RNA regulation (lncRNA) of glycolysis, provides new therapeutic targets for OS. Clinical research also highlights the importance of global participation in clinical trials, with the EuroSarcoma Network’s RECOGNIZE study demonstrating the feasibility and necessity of global participation to improve accessibility to new treatments. Despite these advancements, the treatment of OS remains challenging, and future research should continue to focus on optimizing targeted therapies, refining immunotherapeutic and metabolic interventions, improving early detection methods, integrating precision medicine, and innovating clinical trial designs to effectively translate research findings into better outcomes, ultimately improving the prognosis and quality of life for OS patients.

A recent focus of research has been on the Wnt/β-catenin signaling pathway, which plays a significant role in OS progression. Its activation at the tumor-stroma interface has been shown to facilitate tumor development (Ding and Chen). Additionally, MET, a receptor for hepatocyte growth factor (HGF), is overexpressed in OS and associated with poor prognosis (Zeng et al.). Targeting MET has shown therapeutic potential, and ongoing studies aim to explore combination therapies targeting both MET and other key pathways. Furthermore, novel biomarkers such as APO-TCN2 (Lacinski et al.) and DR5 (Gamie et al.) have been identified, with proteomic and transcriptomic studies indicating their correlation with improved overall survival in OS and other sarcomas. Research should focus on further exploring the interactions between Wnt/β-catenin and other signaling pathways, validating APO-TCN2 and DR5 as biomarkers for early detection, developing more specific MET inhibitors, and investigating combination therapies that could improve treatment outcomes in OS. Moreover, understanding the crosstalk between these pathways and their influence on immune cell recruitment presents new opportunities for therapeutic interventions. Recent advances in single-cell RNA sequencing (scRNA-seq) have enabled a more detailed exploration of TME, providing insights into how immune cells interact with tumor cells and surrounding stromal components, which can help identify novel therapeutic targets (Zou et al., 2022). Additionally, combining CRISPR-Cas9 gene editing technology with nanoparticle drug delivery systems allows for precise manipulation of immune responses and targeted gene editing of immune evasion mechanisms, presenting new avenues for treatment. By modulating immune cell recruitment and activation within the TME, these strategies aim to enhance the efficacy of immunotherapies and offer potential improvements in the treatment of OS and other cancers (Xu et al., 2021). These approaches hold promise for personalized therapy tailored to the unique immune landscape of each patient.

In recent years, immunotherapy has shown promise in treating various cancers, but its efficacy in OS remains limited due to the complexity of the TME. Immune evasion mechanisms, such as the inactivation of phosphatase and tensin homolog deleted on chromosome 10 (PTEN), lead to hyperactivation of the PI3K-AKT pathway, which significantly reduces the effectiveness of immunotherapies (Piro et al., 2019). The tumor cells in OS employ multiple mechanisms to evade immune detection, making single-agent therapies ineffective. Therefore, combination therapies aimed at overcoming tumor heterogeneity and immune evasion mechanisms are being actively researched. Fourth-generation CAR-T cell therapies, which utilize co-expression of CXC chemokine receptor 5 (CXCR5) and interleukin (IL)-7, have demonstrated preclinical efficacy (Xu et al., 2021). One study showed that the co-expression of CXCR5 and IL-7 significantly enhanced CAR-T cell effectiveness by improving tumor penetration, persistence, and cytotoxicity (Hui et al.). Additionally, a bibliometric analysis of immunotherapy research for OS highlights China’s role as a leader in this field, with significant trends pointing toward increasing interest in the TME, immune checkpoint inhibitors, and CAR-T cell therapy (Hui et al.).

The TME plays an integral role in OS metastasis. Recent studies have shown that neutrophil plasticity influences tumor progression, with the N1 subset exhibiting tumoricidal effects and the N2 subset supporting premetastatic niche formation (Yu and Yao, 2024). Targeting these neutrophil subsets could offer a novel strategy to prevent metastasis (Xia et al.). Moreover, metabolic reprogramming, particularly the enhancement of the Warburg effect in OS tumor cells, presents new therapeutic opportunities. Modulating metabolic pathways to inhibit OS cell growth and migration has become an area of intense research (Zeng et al.). lncRNAC1QTNF1-AS1 has been shown to regulate glycolysis through miR-34a-5p, promoting tumor growth and metastasis (Zhang et al.). Targeting these metabolic pathways, especially glycolysis, may provide new therapeutic strategies to treat OS.

One of the major challenges in OS research is the limited representation of pediatric populations in clinical trials. Efforts to establish decentralized clinical trial networks, such as the EuroSarcoma Network’s RECOGNIZE study, have proven that global participation is not only feasible but also critical for ensuring equitable access to new therapies. These efforts can serve as a model for future trials aiming to enhance diversity and ensure that all patient populations benefit from treatment advancements (Hu et al.). In addition, a clinical case report describes a rare occurrence of costal chondrosarcoma secondary to multiple hereditary exostoses (HME), which compressed the right ventricle. The patient underwent intralesional resection followed by adjuvant radiotherapy, with no recurrence observed during the one-year follow-up. This case highlights the importance of early imaging screening and multidisciplinary management in patients with HME to improve diagnostic accuracy, optimize treatment strategies, and reduce the risk of recurrence (Yang et al.).

As research in molecular biology and immunology progresses,OS treatment is entering an innovative phase. Future studies should prioritize precision medicine and individualized treatment plans, with a particular focus on integrating immunotherapy and metabolic interventions. Further exploration of lncRNA-miRNA interactions in OS is essential to identify new therapeutic targets, particularly those involving glycolytic enzymes (Zhang et al.). A review of competing endogenous RNA (ceRNA) mechanisms in cancer underscores the importance of ceRNA interactions in cancer biology, which could open new avenues for targeted therapy (Cont et al., 2021). Additionally, addressing immune resistance mechanisms and tumor resistance will be crucial in advancing OS treatment. Enhancing early screening methods for detecting malignant transformations in HME and refining treatment protocols for rare conditions such as costal chondrosarcoma will also be essential. The focus should be on translating these innovations into clinical practice to improve patient outcomes and inform personalized therapeutic approaches. By fostering interdisciplinary collaboration and embracing novel strategies, OS treatment outcomes can be significantly improved, ultimately offering patients better prognoses and quality of life. Integrating multi-omics data—such as genomics, proteomics, and metabolomics—provides a deeper understanding of tumor heterogeneity and the molecular basis of osteosarcoma, facilitating the development of precision medicine strategies tailored to individual patients (Zou et al., 2022). These omics-guided approaches can reveal actionable mutations, dysregulated signaling pathways, and metabolic vulnerabilities, enabling clinicians to select therapies that are more likely to be effective while minimizing unnecessary toxicity. This systems-level approach holds transformative potential for OS management (Sabit et al., 2025; Chadha and Huang, 2022).

This Research Topic underscores the importance of innovative, interdisciplinary approaches in overcoming the current limitations of OS treatment. The integration of novel therapeutic strategies, including targeted metabolic interventions, advances in immunotherapy, and TME modulation, offers new hope for improving patient outcomes. Moreover, addressing structural barriers to clinical trial design—particularly by enhancing global participation and equity—is crucial for ensuring these innovations benefit all affected populations.
